# Protective Role of Eicosapentaenoic and Docosahexaenoic and Their *N*-Ethanolamide Derivatives in Olfactory Glial Cells Affected by Lipopolysaccharide-Induced Neuroinflammation

**DOI:** 10.3390/molecules29204821

**Published:** 2024-10-11

**Authors:** Rosalia Pellitteri, Valentina La Cognata, Cristina Russo, Angela Patti, Claudia Sanfilippo

**Affiliations:** 1 Institute for Biomedical Research and Innovation, National Research Council, Via P. Gaifami 18, I-95126 Catania, Italy; valentina.lacognata@cnr.it; 2 Department of Biomedical and Biotechnological Sciences, Section of Pathology, University of Catania, Via Santa Sofia 97, I-95123 Catania, Italy; cristina.russo@unict.it; 3 Institute of Biomolecular Chemistry, National Research Council, Via P. Gaifami 18, I-95126 Catania, Italy; claudia.sanfilippo@cnr.it

**Keywords:** polyunsaturated fatty acids, *N*-eicosapentaenoyl ethanolamide, *N*-docosahexaenoyl ethanolamide, olfactory ensheathing cells, neuroinflammation, neuroprotection, neurodegenerative diseases

## Abstract

Neuroinflammation is a symptom of different neurodegenerative diseases, and growing interest is directed towards active drug development for the reduction of its negative effects. The anti-inflammatory activity of polyunsaturated fatty acids, eicosapentaenoic (EPA), docosahexaenoic (DHA), and their amide derivatives was largely investigated on some neural cells. Herein, we aimed to elucidate the protective role of both EPA and DHA and the corresponding *N*-ethanolamides EPA-EA and DHA-EA on neonatal mouse Olfactory Ensheathing Cells (OECs) after exposition to lipopolysaccharide (LPS)-induced neuroinflammation. To verify their anti-inflammatory effect and cell morphological features on OECs, the expression of IL-10 cytokine, and cytoskeletal proteins (vimentin and GFAP) was evaluated by immunocytochemical procedures. In addition, MTT assays, TUNEL, and mitochondrial health tests were carried out to assess their protective effects on OEC viability. Our results highlight a reduction in GFAP and vimentin expression in OECs exposed to LPS and treated with EPA or DHA or EPA-EA or DHA-EA in comparison with OECs exposed to LPS alone. We observed a protective role of EPA and DHA on cell morphology, while the amides EPA-EA and DHA-EA mainly exerted a superior anti-inflammatory effect compared to free acids.

## 1. Introduction

Most degenerative diseases, such as Alzheimer’s disease, Parkinson’s disease, Huntington’s disease, and multiple sclerosis, are associated with neuroinflammation, which is characterized by glia activation (microglia and astrocytes), resulting in the release of several soluble factors, including cytokines and chemokines [1,2]. These molecules are responsible for neuroinflammation and oxidative stress, two critical processes linked to neurodegenerative diseases. In addition, they are able to induce neurotoxicity and neurodegeneration and to alter neurotransmission [3,4]. The initial acute inflammation can turn into chronic, leading to tissue destruction and cell death caused by the macrophage and lymphocyte influx coming from the circulatory district, as observed in spinal cord lesions [5]. Guo et al. [6] reported that microglia, depending on micro-environmental modifications, may act both as pro-inflammatory and anti-inflammatory, inducing anti-inflammatory and neuroprotective effects. Indeed, pro-inflammatory microglia produce lactate, which promotes the release of cytokines and interleukins [7,8], thus driving neuroinflammation, but on the other hand, it plays a role in resolving it and in repairing lesions of the nervous system [9].

In recent years, a growing interest has been directed towards the development of active drugs for the reduction or elimination of neuroinflammation effects, considered as symptoms in the onset of different neurodegenerative diseases. Eicosapentaenoic acid (EPA, 20:5, *n*-3) and docosahexaenoic acid (DHA, 22:6, *n*-3), belonging to the class of *n*-3 long-chain polyunsaturated fatty acids (PUFA), are known as important constituents (especially DHA) of brain phospholipids and play a critical role in the structure and functions of neuronal membranes [10,11]. In fact, the brain is the organ richest in lipids after adipose tissue [12]. A difference between EPA and DHA consists in their distribution in the brain: DHA is present at a high concentration within the brain compared with EPA, and both are able to cross the blood–brain barrier [13]. Some authors reported that when rat hippocampal culture medium was added with DHA, an increase in both neurite length and the number of neuronal branches was observed [11]. Other studies have highlighted that DHA stimulates the release of brain-derived neurotrophic factor (BDNF) and is able to control morphology and synaptogenesis [14,15], so acting as a potential therapeutic agent in neurodegenerative diseases [16]. These observations suggest an important relation between DHA deficit and the onset of neurodegenerative diseases [17].

A plethora of studies based on *n*-3 PUFA dietary supplementation have provided converging evidence that alterations in PUFA levels and metabolism may affect the control of neuroinflammation and related degenerative processes [18,19]. In vitro experiments on microglial cells exposed to lipopolysaccharide (LPS)-induced inflammation have evidenced that free *n*-3 PUFA displays dose-dependent anti-inflammatory activity, exerted by means of different mechanisms such as the inhibition of NO synthase and COX-2 inflammatory enzymes, the reduction in the expression of matrix metalloproteinase (MMP-9) and the decrease in the production of proinflammatory cytokines [20,21,22].

Through oxidative enzymatic pathways, PUFA is converted into specific derivatives, such as resolvins, protectins, and maresins, which have emerged as “specialized pro-resolving mediators (SPM)” of inflammation [23,24], while their conjugation with amines leads to PUFA-amides that exert their effects through interaction with cannabinoid receptors CB1 and CB2 [25]. Increased levels of docosahexaenoyl ethanolamide (DHA-EA) have been detected in the brains of animals fed with a DHA/EPA-supplemented diet, and its role in the suppression of neuroinflammation in mice has been related to the enhancement of cAMP/PKA signaling and the inhibition of NF-κB activation [26]. 

In spite of mounting evidence of beneficial influences of *n*-3 PUFA and related derivatives, the elucidation and comparison of specific effects due to individual *n*-3 PUFA compounds is rather limited [13] and deserves focused investigation. In this context, we planned to individually test EPA and DHA, as well as the corresponding *N*-ethanolamides EPA-EA and DHA-EA, for their protective effect on Olfactory Ensheathing Cells (OECs) exposed to LPS-induced neuroinflammation.

OECs constitute an unusual population of glial cells present in the Olfactory System, sharing properties with both Schwann cells of the Peripheral Nervous System (PNS) and astrocytes of the Central Nervous System (CNS). They accompany the small nonmyelinated axons of the Olfactory Receptor Neurons along their whole length, from the basal lamina of the epithelium to the olfactory bulb, crossing the transitional zone between the PNS and CNS [27,28,29]. The OECs are a source of different growth factors [29,30,31], also expressing adhesion molecules [32] and numerous markers [29], including nestin [33], which is a specific stem cell marker. For their properties, they are able to stimulate axonal regeneration in the lesioned sites of the Nervous System, restoring functional connections and promoting vascularization and remyelination of damaged axons [34,35]. Another very important peculiarity of OECs consists in their ability to intermingle with astrocytes when transplanted, unlike Schwann Cells, which instead stimulate astrocytic gliosis and cystic cavitation in damaged sites [36]. Furthermore, many authors have highlighted the phagocytic activity of OECs, which is useful for removing necrotic cells and stimulating neural regrowth [36,37], and it has been demonstrated that these cells are able to produce anti-inflammatory molecules regulating, when transplanted, inflammation localized in the injured spinal cord [38]. In addition, in a previous study, we have shown that OECs express ghrelin, a gut-brain peptide hormone with anti-inflammatory, antioxidant, and anti-apoptotic properties, and its receptor [39].

Therefore, contrasting neuroinflammation in OECs could offer a novel therapeutic target for neurological diseases, and we planned to investigate the potential protective role of EPA and DHA, as well as the corresponding *N*-ethanolamides EPA-EA and DHA-EA, for the first time in OECs. In all the experiments, both OECs alone and OECs exposed to LPS-induced neuroinflammation for 24 h were treated with the target compounds, while untreated OECs but LPS-stressed were used as a reference for the cytotoxic effects induced by inflammation. In the first instance, the treatment-induced changes in cell morphology and viability were assessed. Then, since apoptosis and mitochondrial dysfunctions play an important role in the physiological development of the nervous system, brain aging, and the onset and progression of several neurodegenerative diseases [40], mitochondrial toxicity and apoptosis levels were evaluated by specific tests (MitoHealth and TUNEL assays, respectively). Furthermore, the expression of some proteins, whose increased production is associated with neuroinflammation, was evaluated by immunocytochemical procedures. In this context, we considered IL-10, which is one among many cytokines produced by LPS induction, and Glial Fibrillary Acidic Protein (GFAP) and vimentin, two cytoskeleton markers indicating glial reactivity, as it is known that LPS is a glial activator for the inflammatory induction in neural cells with microglia activation [41]. 

Here, we report the obtained results that evidenced positive effects exerted by EPA, DHA, EPA-EA, and DHA-EA in contrasting neuroinflammation, in some cases with chemical structure-related differences. 

## 2. Results

### 2.1. Chemical Compounds

**Scheme 1:**
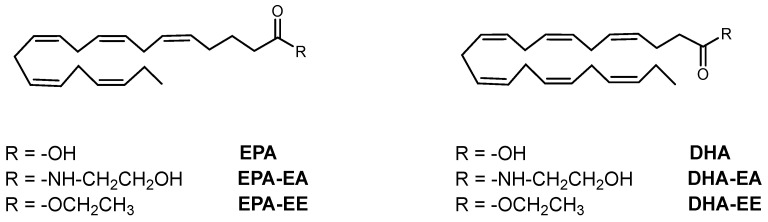
Chemical structures of the compounds tested in this study and their synthetic precursors.

While free acids, EPA and DHA, are commercially available, the corresponding amides were prepared by aminolysis of the ethyl esters of EPA and DHA with ethanolamine in the presence of immobilized lipase from *Candida antarctica* (Novozym 435) and molecular sieves in tert-butyl methyl ether. This biocatalyzed procedure offers advantages in mild reaction conditions and complete chemoselectivity toward the formation of amides over esters, resulting in a high yield of the target products EPA-EA and DHA-EA [42]. The amides were checked for their purity (>98%) by HPLC and 1H-NMR analysis immediately before their dissolution in DMSO for biological assays (Scheme 1).

### 2.2. Morphological Features of OECs after Treatments

**Figure 1:**
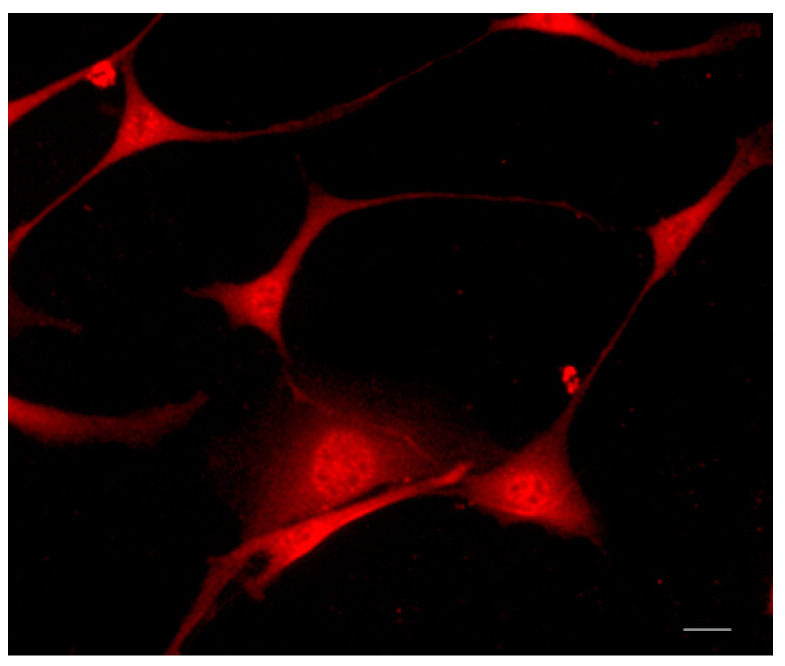
Immunofluorescent staining for S-100 protein expression in primary mouse OECs. Magnification: 20×. Scale bar: 10 µm.

Our study was focused on exposing OECs to LPS to induce an inflammatory event. In physiological conditions, the majority of OECs exhibited both star and spindle shapes (Figure 1), which are typical morphological features of in vitro OECs [29,33]. 

**Figure 2:**
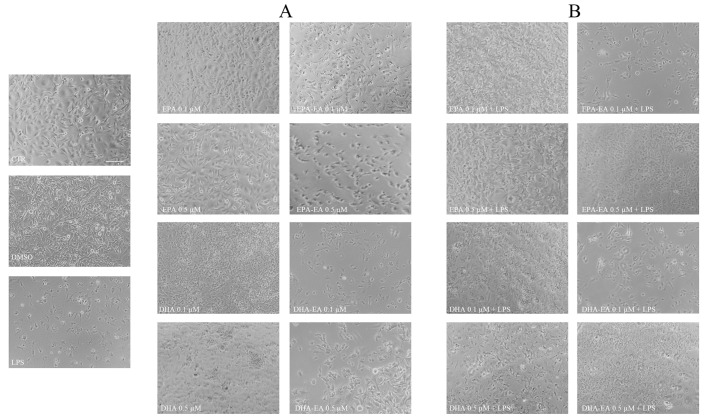
Morphological analysis of OECs by phase contrast microscopy. Representative fields of OECs, without (**A**) and with (**B**) LPS exposure, were treated with EPA, DHA, EPA-EA, and DHA-EA at different concentrations (0.1 µM and 0.5 µM) for 24 h. Magnification: 10×. Scale bars: 50 μm.

To control morphological changes induced by the different treatments, the cells were monitored by phase-contrast inverted microscopy, and by this way, it was evidenced that EPA, DHA, and EPA-EA did not promote morphological variations at the tested concentrations, while DHA-EA at both concentrations caused some cell suffering and a reduction in cell number if compared with the controls (Figure 2A), as also observed following exposure to LPS. 

When OECs were exposed to LPS and treated with EPA or DHA, the examination of the cell morphological features revealed a protective effect at both tested concentrations. On the contrary, EPA-EA and DHA-EA protected LPS-exposed OECs only at higher concentrations, while at lower concentrations, it seemed that the cells lost their usual morphology (Figure 2B).

### 2.3. Cell Viability (MTT Test)

**Figure 3:**
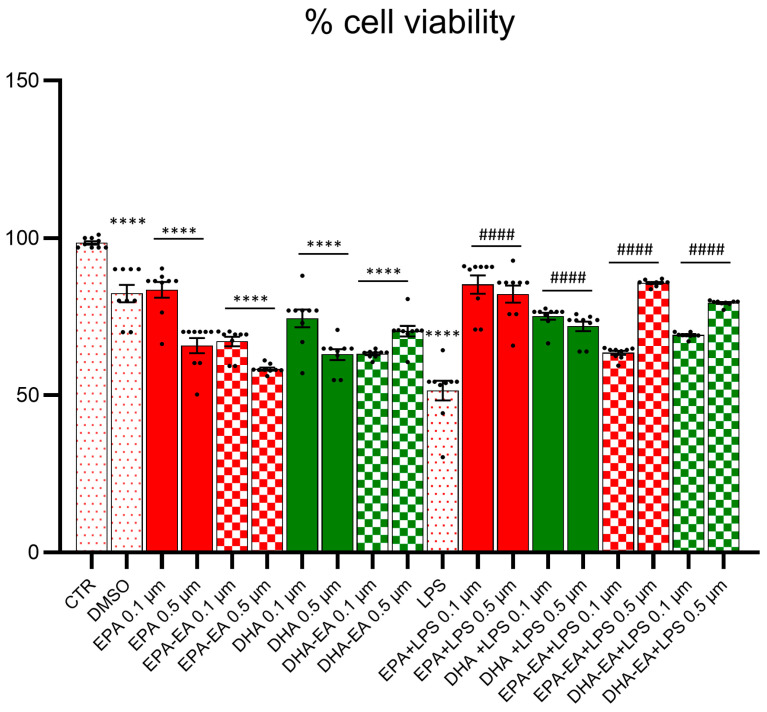
Percentage of viable OECs after treatment with EPA, DHA, EPA-EA, and DHA-EA. OECs without or with LPS stress were treated with EPA, DHA, EPA-EA, and DHA-EA at different concentrations (0.1 µM and 0.5 µM) for 24 h. One-way ANOVA experimental groups vs. CTR, F (18, 152) = 37.60. One-way ANOVA experimental groups vs. LPS, F (16, 136) = 28.58. Dunnett’s multiple comparison test: **** *p* < 0.0001 vs. CTR; #### *p* < 0.0001 vs. LPS.

In all experimental conditions, the MTT test was performed in order to evaluate the cell viability. The optimal concentrations of EPA, DHA, and the corresponding amides, as well as the suitable time of exposure, were chosen after a set of preliminary experiments. As shown in Figure 3, LPS was able to decrease the percentage of cell viability by almost 50% compared to control and DMSO, the latter used to solubilize the tested compounds. When the OECs were treated with EPA or DHA, better cell viability was determined at 0.1 µM for both molecules compared to 0.5 µM, and the treatment with EPA protected the LPS-exposed OECs more incisively than DHA in both the tested concentrations. Treatment with amides EPA-EA and DHA-EA decreased cell viability compared with the controls (CTR and DMSO), with a major incidence at 0.5 µM for EPA-EA and at 0.1 µM for DHA-EA. When OECs were exposed to LPS, the treatment with all the tested compounds resulted in sensibly higher cell viability compared to the control, and EPA at 0.1 µM concentration was the most effective. The treatment with DHA led to lower cell viability compared to EPA at both the tested concentrations, while EPA-EA and DHA-EA showed similar outcomes in protecting LPS-exposed OECs, with higher activity at 0.5 µM concentration. These data are comparable with those obtained by phase-contrast inverted microscopy. 

### 2.4. Influence on OEC Apoptosis (TUNEL Assay)

**Figure 4:**
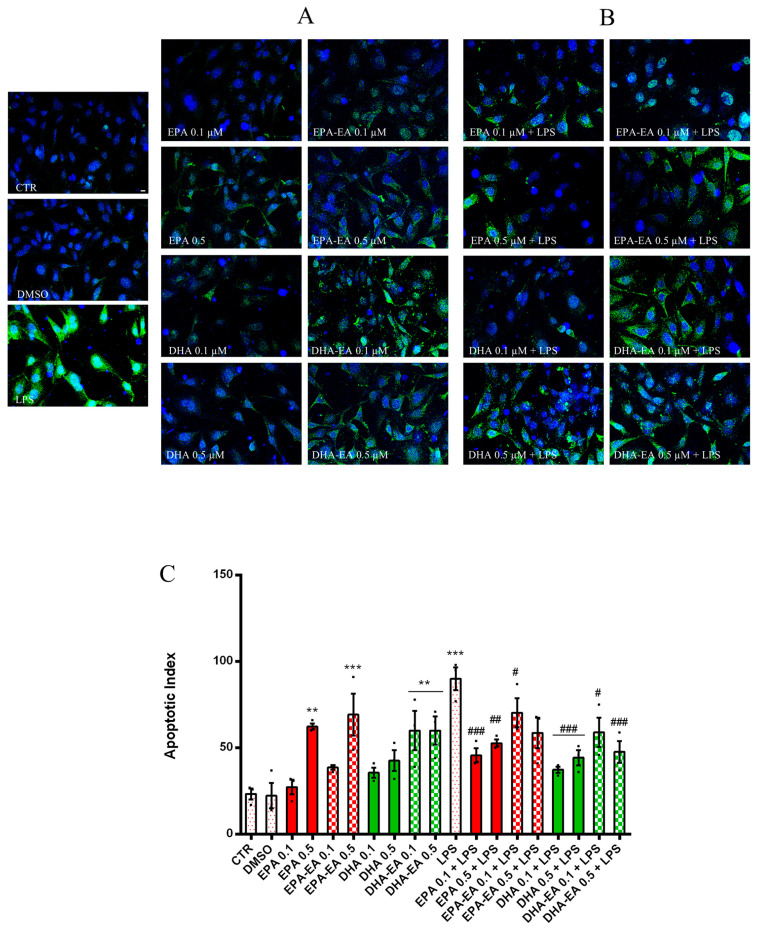
Dead cell detection by TUNEL assay. The test was carried out on OECs without (**A**) and with (**B**) LPS exposure and treated with EPA, DHA, EPA-EA, and DHA-EA. Photomicrographs are representative of randomly selected fields and were scanned by a Nikon Ti Eclipse inverted microscope. Scale bar 10 μm. (**C**) Data were taken from at least three independent experiments. TUNEL-positive cells were calculated as described in Section 4 and are reported as mean ± SEM. One-way ANOVA experimental groups vs. CTRL condition F (10, 22) = 9.913; *p* < 0.0001; One-way ANOVA experimental groups vs. LPS treatment F (8, 18) = 6.616; *p* = 0.0004; Dunnett’s multiple comparison tests: ** *p* < 0.01 and *** *p* < 0.001 vs. Ctrl; # *p* < 0.05, ## *p* < 0.01 and ### *p* < 0.001 vs. LPS.

To evaluate the death of OECs exposed to LPS and treated with EPA or DHA, we used the TUNEL assay, based on the incorporation of modified nucleotide dUTPs by the enzyme terminal deoxynucleotidyl transferase (TdT) at the 3′-OH ends of fragmented DNA. The exposure to LPS for 24 h determined a significant increase in TUNEL-positive cells, denoting the presence of a higher amount of fragmented DNA and higher death levels if compared to controls (CTR and DMSO). The treatment with EPA and EPA-EA at 0.1 µM and DHA at both 0.1 µM and 0.5 µM did not cause significant changes in the number of dead cells compared to controls (Figure 4A,C). When OECs were exposed to LPS, the treatment with EPA or DHA in both concentrations reduced the levels of fragmented DNA, decreasing the number of TUNEL-positive cells. This result was more evident for LPS-exposed OECs treated with DHA at 0.1 µM concentration (Figure 4B,C).

### 2.5. Mitotoxicity and Cytotoxicity of LPS in OECs (MitoHealth Assay)

**Figure 5:**
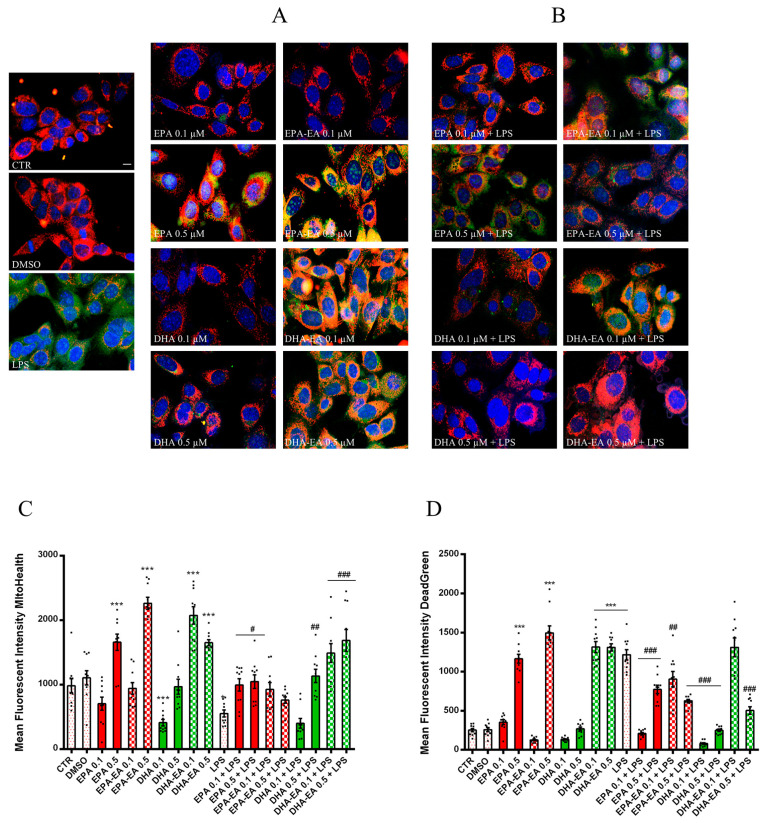
Mitotoxicity and cytotoxicity levels in OECs exposed to different treatments. The test was carried out on OECs without (**A**) and with (**B**) LPS exposure and treated with EPA, DHA, EPA-EA, and DHA-EA. Representative images were taken from randomly selected fields of slides scanned by a Nikon Ti Eclipse inverted microscope (scale bar 10 μm). Mean Fluorescent Intensity (MFI) values (mean ± SEM) of the ratio MitoHealth (**C**) and DeadGreen (**D**) were calculated as described in the Material and Methods Section. Values are reported as mean ± SEM. For Mitohealth staining, One-way ANOVA experimental groups vs. CTRL condition F (10, 99) = 38.81; *p* < 0.0001; One-way ANOVA experimental groups vs. LPS treatment F (8, 81) = 14.69; *p* < 0.0001. For DeadGreen staining, One-way ANOVA experimental groups vs. CTRL condition F (10, 99) = 137.8; *p* < 0.0001; One-way ANOVA experimental groups vs. LPS treatment F (8, 81) = 49.71; *p* < 0.0001. Dunnett’s multiple comparison test: *** *p* < 0.001 vs. CTRL # *p* < 0.05, ## *p* < 0.01, and ### *p* < 0.001 vs. LPS.

To investigate the protective effects of tested compounds in OECs exposed to LPS, we used a double-stain-based commercial kit able to simultaneously measure both mitotoxicity and cytotoxicity. In particular, the MitoHealth red stain accumulated in mitochondria in live cells proportionally to the mitochondrial membrane potential, and Image-iT^®^ DEAD™ Green penetrates inside cells when plasma membrane integrity is compromised, so measuring the cytotoxicity. We found that incubation of OECs with LPS determined a significant decrease in MitoHealth staining and an increase in DeadGreen accumulation against controls (Figure 5), as the exposed OECs exhibited lower mitochondrial membrane potential and reduced membrane integrity compared with controls. Our results show that LPS causes mitochondrial malfunction and the formation of green fluorescent nucleic acid aggregates at the nuclear level. The treatment of OECs with EPA (acids or amides) at 0.5 µM concentration and DHA-EA at both concentrations determined a significant activation of mitochondrial activity, which was maintained for EPA and DHA at 0.5 µM and DHA-EA at both concentrations (Figure 5A,C) also when cells were exposed to LPS (Figure 5B,C). The cytotoxicity evaluated by DeadGreen accumulation (Figure 5D) was in accordance with TUNEL results (Figure 4C).

### 2.6. IL-10 Expression by Immunofluorescence

**Figure 6:**
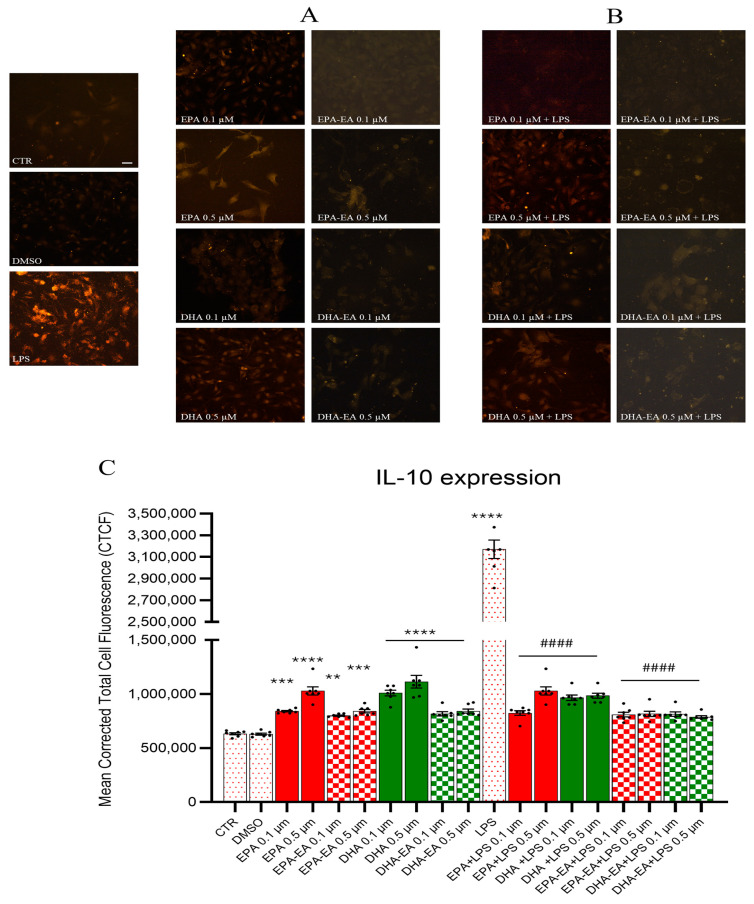
Immunocytochemistry for IL-10 in OECs exposed to different treatments. The test was carried out on OECs without (**A**) and with (**B**) LPS exposure and treated with EPA, DHA, EPA-EA, and DHA-EA. Immunostained samples were analyzed with a Zeiss fluorescence microscope, and images were captured with the Axiovision imaging system. (**C**) Fluorescence quantification data for IL-10 in each OEC cultured in different conditions. Bars represent CTCF mean value ± SD, obtained from at least three independent experiments. One-way ANOVA experimental groups vs. CTR, F (18,114) = 292.8. One-way ANOVA experimental groups vs. LPS, F (16, 102) = 278.5. Dunnett’s multiple comparison test: ** *p* < 0.01; *** *p* < 0.001; **** *p* < 0.0001 vs. CTR; #### *p* < 0.0001 vs. LPS. Scale bar 20 μm.

The expression of the anti-inflammatory marker IL-10 in the OECs grown in all the different conditions was evaluated through the immunofluorescence technique, and the experimental model was effective in demonstrating that LPS was able to stimulate an inflammatory activity. The treatment of OECs with all the considered compounds did not give any strong evidence of inflammation (Figure 6A), even if a slightly enhanced positivity was revealed by the fluorescence quantification (Figure 6C). A strong positivity to IL-10 was found in OECs exposed to LPS, but in the presence of EPA, DHA, EPA-EA, and DHA-EA at both concentrations, the labeling sensibly decreased, indicating an active role of the tested compounds in protecting the cells from LPS-induced neurotoxic effects. (Figure 6B). More in detail, the fluorescence quantification (Figure 6C) evidenced that in the presence of EPA-EA or DHA-EA, the same levels of IL-10 expression as in control were found in OECs, both non-stressed and stressed with LPS.

### 2.7. GFAP and Vimentin Expression by Immunofluorescence

To identify glial reactivity in OECs and related changes in morphological features, the expression of some cytoskeleton markers, such as GFAP and vimentin, was evaluated by immunocytochemistry in cells treated with EPA, DHA, EPA-EA, and DHA-EA at both concentrations (0.1 µM and 0.5 µM) and exposed or not to LPS. 

**Figure 7:**
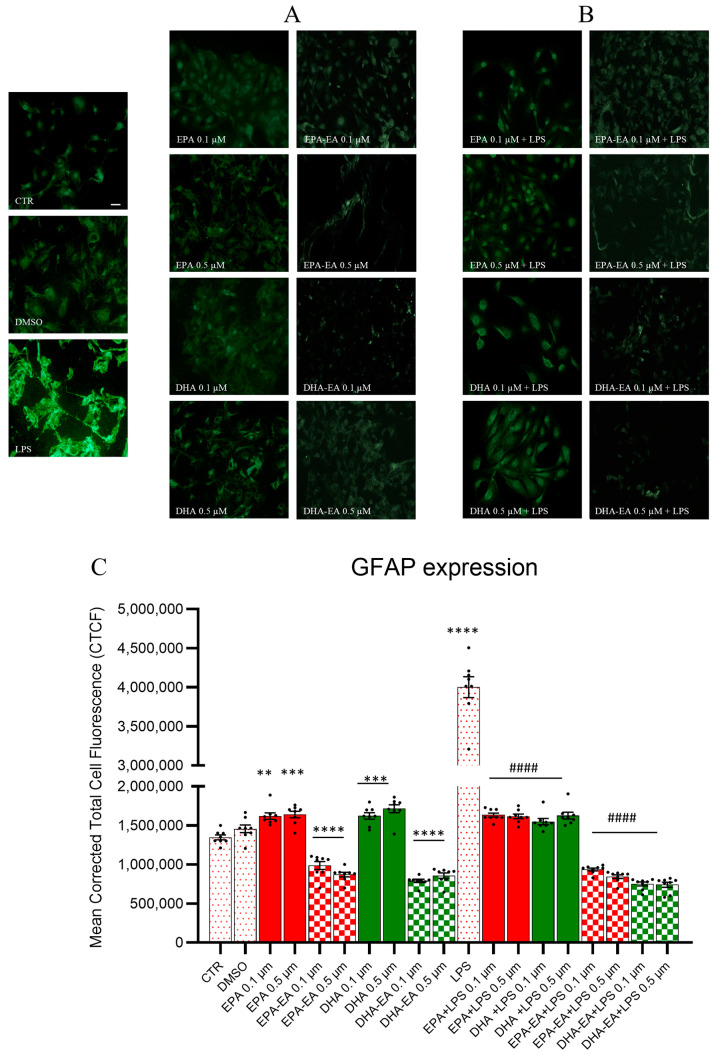
Immunocytochemistry for GFAP in OECs exposed to different treatments. The test was carried out on OECs without (**A**) and with (**B**) LPS exposure and treated with EPA, DHA, EPA-EA, and DHA-EA. (**C**) Fluorescence quantification data for GFAP in each OEC treatment with EPA, DHA, EPA-EA, and DHA-EA at different concentrations (0.1 µm and 0.5 µm). Bars represent CTCF mean value ± SD, obtained from at least three independent experiments. One-way ANOVA experimental groups vs. CTR, F (18, 133) = 101.5. One-way ANOVA experimental groups vs. LPS, F (16, 119) = 116.5. Dunnett’s multiple comparison test: ** *p* < 0.01; *** *p* < 0.001; **** *p* < 0.0001 vs. CTR; #### *p* < 0.0001 vs. LPS. Scale bar 20 μm.

**Figure 8:**
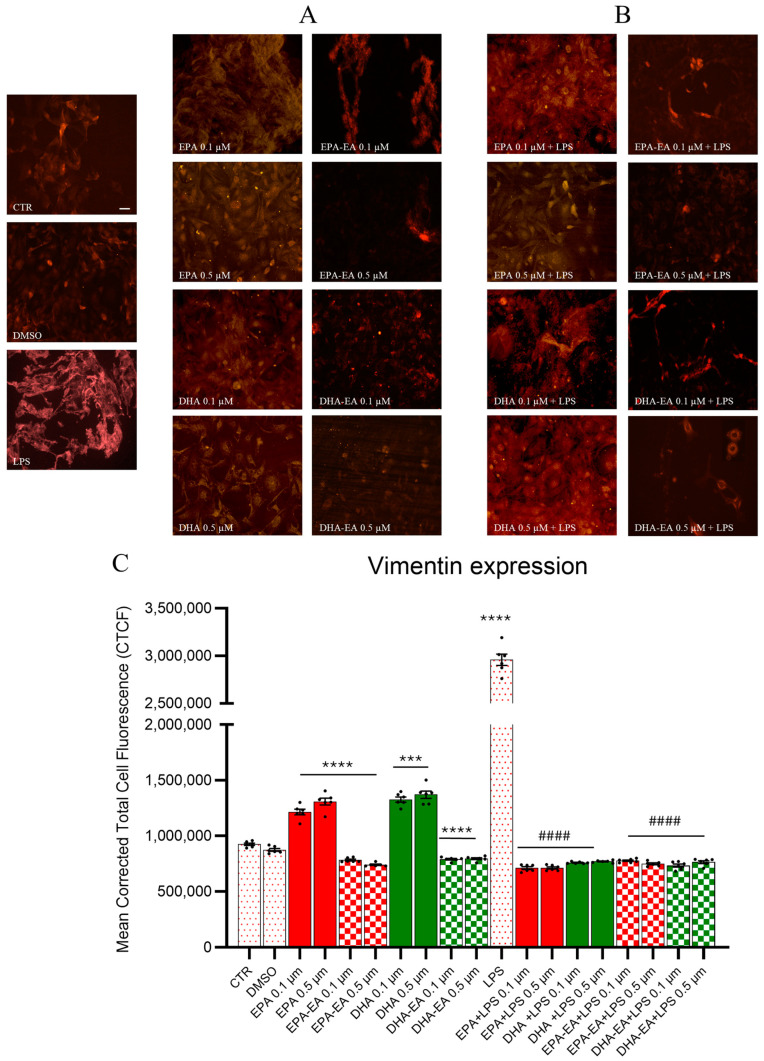
Immunocytochemistry for vimentin in OECs exposed to different treatments. The test was carried out on OECs without (**A**) and with (**B**) LPS exposure and treated with EPA, DHA, EPA-EA, and DHA-EA. (**C**) Fluorescence quantification data for vimentin in each OEC treatment with EPA, DHA, EPA-EA, and DHA-EA at different concentrations (0.1 µm and 0.5 µm). Bars represent CTCF mean value ± SD, obtained from at least three independent experiments. One-way ANOVA experimental groups vs. CTR, F (18, 95) = 375.5. One-way ANOVA experimental groups vs. LPS, F (16, 85) = 396.1. Dunnett’s multiple comparison test: *** *p* < 0.001; **** *p* < 0.0001 vs. CTR; #### *p* < 0.0001 vs. LPS. Scale bar 20 μm.

As shown in the control samples of Figure 7 and Figure 8, a strong positivity for both proteins, GFAP and vimentin, was found in OECs exposed to LPS with respect to controls, as the inflammatory activity induces the activation of the glia cells. The treatment of OECs with the tested compounds showed some increase in vimentin expression for EPA and DHA but not for EPA-EA and DHA-EA, as better evidenced by fluorescence quantification (Figure 7A,C). When the same test was carried out for the detection of GFAP expression, no substantial difference was found between EPA and DHA treatment compared to the non-treated OECs (Figure 8A,C). 

From images shown in Figure 7B and Figure 8B and the corresponding histograms for fluorescence quantification (Figure 7C and Figure 8C), it can be evidenced that the positivity of GFAP and vimentin in OECs cells, as such or exposed to LPS, treated with EPA-EA and DHA-EA was lower compared to that observed for EPA and DHA.

## 3. Discussion

In this study we investigated, for the first time, the role of EPA and DHA and corresponding *N*-ethanolamides EPA-EA and DHA-EA in OECs exposed to stress of LPS for 24 h and reported their protective activity against cell death and inflammation.

Several works have reported that PUFA is essential for the normal development of the central nervous system and for neural stem cell differentiation [43]. In particular, DHA plays a role in neuropsychiatric disorders, such as depression or dementia [44], and it has been demonstrated that PUFA deficiency can affect cerebral functions and alter the development of the brain, involving the membranes of neurons, oligodendrocytes, and astrocytes [12].

In this study, we chose to use mouse primary cultures of OECs, a particular glial cell type located in the olfactory system, as a useful experimental model for neuroinflammation after exposing them to LPS to induce an inflammatory event. Due to their peculiar characteristics, in recent years, OECs have attracted interest for their use in cell therapy [33,35,45], and LPS is considered an effective insult for inflammatory induction in neural cells. The concentrations of the compounds and DMSO used in all the experiments were preliminarily assessed as not toxic for the cells. 

The evaluation of protective effects exerted by EPA, DHA, and the related ethanolamides EPA-EA and DHA-EA on viability and cellular morphology of OECs exposed to LPS-induced inflammation was carried out by both toxicity and immunocytochemistry assays. 

Results from MTT, MitoHealth, and TUNEL assays all evidenced, for the first time, the efficacy of the tested compounds in improving OEC viability and contrasting the cytotoxic effect exerted by LPS on these cells. A similar protective effect had also been observed in a model set up with differentiated SH-SY5Y cells exposed to Aβ25–35 [46]. More in detail, we found that EPA exerts greater protection than DHA on OECs exposed to LPS, in agreement with data reported by Ceccarini et al. [14] that demonstrated that EPA, but not DHA, reduces the toxic effect exerted by neurotoxin 6-hydroxydopamine (6-OHDA) in SH-SY5Y cell line, through increase BNDF and GDNF levels via epigenetic mechanisms.

Apoptosis has been found to be responsible for pathological conditions for the loss of neurons in several neurodegenerative diseases, such as Parkinson’s disease, Alzheimer’s disease, amyotrophic lateral sclerosis, and aging. Brains of patients suffering neurodegenerative diseases have shown significant DNA fragmentation detected by the TUNEL test, reduced expression of anti-apoptotic Bcl-2, and the presence of activated caspase-3 in neurons, so demonstrating the involvement of apoptosis in the pathogenesis of neurodegenerative diseases [47]. Mitochondrial fragmentation induced by the expression of caspase-3-cleaved tau sensitizes mitochondria to a neurotoxic effect associated with Alzheimer’s disease [48]. Furthermore, the role of mitochondria, in addition to providing energy through ATP, is to regulate cell calcium activity, which is essential for neurotransmitter release [49]. Thus, we decided to highlight the potential protective role of the target compounds by monitoring their efficacy in reducing apoptosis and improving the mitochondrial health of LPS-exposed OECs. 

The obtained results by MitoHealth and TUNEL assays for EPA and DHA (both acids and amides) on OECs suggest a beneficial effect of the tested compounds in controlling effects of inflammation and in preventing the onset of neurodegenerative diseases through a reduction in mitochondrial fragmentation and apoptosis, in agreement with Eckert et al. [50]. 

Some authors report that cytoskeleton proteins, such as vimentin and GFAP, are involved in several cellular functions and in stress conditions, so their expression is considered a biomarker of astrocyte activation and astrogliosis caused by lesions or neurodegenerative diseases [51,52]. Therefore, in order to gain better evidence of the anti-inflammatory activity of the tested compounds, immunocytochemistry assays targeted to the quantification of the expression levels of specific proteins related to inflammation were carried out, and among the possible proteins, we considered two cytoskeleton proteins, vimentin and GFAP, and the cytokine IL-10. Cytoskeleton changes, indeed, are involved in the pathogenesis of some neurodegenerative diseases, and morphological features resulting from increased glial reactivity in OECs exposed to LPS and treated with the compounds in hands were monitored through the expression of cytoskeleton markers, which play an important role in astrogliosis, a typical sign of an inflammation event [53]. 

The treatment of LPS-stressed OECs with EPA, DHA, EPA-EA, and DHA-EA resulted in the same protein expression levels compared to the control, or even lower in the case of GFAP, evidencing an anti-inflammatory activity of these compounds. The decreased GFAP and vimentin expression, indeed, indicated a decrease in reactive LPS-induced astrogliosis and highlighted the protective effect of EPA-EA and DHA-EA on OECs.

The obtained results are in agreement with a study conducted by Tyrtyshnaia et al. [54], in which the authors reported that both EPA-EA and DHA-EA counteract the LPS-mediated increase in GFAP expression, with a superior anti-inflammatory activity found for DHA-EA compared to EPA-EA in vitro and in vivo studies. The lower EPA-EA activity might be due to a lower content of this molecule within the brain compared with DHA-EA [25].

Other support for the anti-inflammatory activity of the tested compounds on OECs comes from the evaluation of levels of IL-10, one among many cytokines produced by LPS induction. While a strong positivity to IL-10 was found in OECs exposed to LPS, levels of IL-10 in treated cells were reduced as in the control. 

These obtained data suggest a structure-related activity, with EPA and DHA predominantly exerting a protective role on cell morphology and viability, as deduced by monitoring obtained from the inverted microscope as well as MTT, TUNEL, and DeadGreen staining in the MitoHealth test, while amides EPA-EA and DHA-EA mainly show anti-inflammatory effects superior to those of free acids due to their ability to better suppress the expression of cytoskeleton proteins and the cytokine IL-10 in LPS-stressed OECs.

Concentration levels of DHA-EA and EPA-EA are related to those of DHA and EPA and have been shown to increase after consumption of a fish oil-rich or DHA-supplemented diet [55]. Therefore, the administration of a DHA/EPA diet could counteract neuroinflammation effects and improve cognitive activities, preventing the decline associated with aging in humans.

## 4. Materials and Methods

### 4.1. Reagents

(5*Z*,8*Z*,11*Z*,14*Z*,17*Z*)-Eicosapentenoicacid (EPA), (4*Z*,7*Z*,10*Z*,13*Z*,16*Z*,19*Z*)-docosa-hexaenoic acid (DHA), and ethanolamine were purchased from Sigma-Aldrich (St. Louis, MO, USA) and used as received. The corresponding ethyl esters EPA-EE and DHA-EE were obtained from Solutex (Madrid, Spain). Immobilized lipase from *Candida antarctica* (Novozym^®^ 435) was obtained from Strem Chemicals Inc. (Newburyport, MA, USA). Additionally, 3Å Molecular sieves (Aldrich, St. Louis, MO, USA) were activated by heating at 100 °C for 3 h before use. Lipopolysaccharide (LPS) was obtained from Santa Cruz (Dallas, TX, USA, sc-3535) and dissolved in sterile PBS. 

### 4.2. Synthesis of EPA-EA and DHA-EA

The synthesis of title compounds was carried out as previously reported [42]. Briefly, to a solution of EPA-EE or DHA-EE (200 mg) in tert-butyl methyl ether (6 mL), Novozym^®^ 435 (100 mg), molecular sieves (100 mg), and ethanolamine (1.1 eqv.) were added, and the suspension stirred in a shaker at 45 °C for 12–24 h. At the end of the reaction, as stated by TLC analysis (*n*-hexane/EtOAc 9:1), the mixture was extracted with 2 M HCl, and the organic phase was separated. The organic solvent was removed under reduced pressure, and the products EPA-EA or DHA-EA were recovered as pale-yellow oils in 90–95% yield. Further purification was carried out by column chromatography on Si gel, eluting with *n*-hexane/EtOAc 1:1 containing 5% EtOH *v*/*v*. ^1^H- and ^13^C-NMR spectra were registered on a Bruker Avance TM 400 spectrometer (Bruker Milano, Milan, Italy) at 400.13 and 100.62 MHz, respectively, and were found in agreement with those reported. Chemical purity (>98%) was checked by HPLC on a SphereClone Silica 80 Å (Phenomenex, Inc. Bologna, Italy) column (250 × 4.6 mm, 5 μm) eluting with *n*-hexane:2-PrOH 85:15 mixture at flow 0.5 mL/min and using UV-detection at 220 nm.

### 4.3. OEC Cultures

OECs were obtained from 2-day-old mouse pups (P2) olfactory bulbs (Envigo RMS s.r.l., Udine, Italy). All the experiments were carried out according to the Italian Guidelines for Animal Care (D.L. 116/92 and 26/2014), which are in compliance with the European Communities Council Directives (2010/63/EU) and approved by the Ethical Committee at the Catania University (Italy) and National Ministry of Health (permit number 174/2017-PR).

Olfactory bulbs were digested in MEM-H, containing a collagenase and trypsin mixture as previously described [56]. Trypsinization was stopped by adding DMEM supplemented with 10% FBS (DMEM/FBS, GIBCO, Waltham, MA, USA). Cells were re-suspended and plated in flasks fed with complete DMEM/FBS. An antimitotic agent, cytosine arabinoside (10−5 M), was added 24 h after initial plating to reduce the number of dividing fibroblasts. When OECs were confluent, they were removed by trypsin, transferred on 25 cm2 flasks, cultured in DMEM/FBS, and incubated at 37 °C in a humidified 5% CO_2_–95% air mixture. 

### 4.4. Treatment of Cells

Purified OECs were grown in DMEM/FBS on both 13 mm diameter glass coverslips and 96-multi-wells plates and incubated at 37 °C in a humidified 5% CO_2_—95% air mixture. In preliminary experiments, we screened different concentrations (0.1, 0.25, 0.5, and 5 µM) of target compounds and LPS (10 and 20 µg/mL) in order to establish non-toxic concentrations. Working concentrations of target compounds were set at 0.1 and 0.5 μM, and no difference was found at higher concentrations (by MTT assay). The optimal time of exposure of OECs to LPS and to the target compounds was set to 24 h, chosen according to our previous study [57]. OEC cultures were divided into different groups: as controls, we considered (1) a group contained only OECs, (2) a group treated with DMSO (0.008% *v*/*v*), the solvent used to solubilize the tested compounds, and (3) a group stressed for 24 h with LPS (10 µg/mL) [58]. In addition, we set up: (4) two groups treated with EPA 0.1 µM or 0.5 µM for 24 h; (5) two other groups treated with DHA 0.1 µM or 0.5 µM for 24 h; (6) two groups treated with EPA-EA 0.1 µM or 0.5 µM for 24 h; (7) other two groups treated with DHA-EA 0.1 µM or 0.5 µM for 24 h. Four other groups were the same as groups 4–7 with the addition of LPS. 

Possible changes in cellular morphology were qualitatively evaluated after 24 h by phase-contrast inverted microscopy (AXIOVERT 100, Zeiss, Jena, Germany), capturing the images with the Axiovision imaging system. For the following experiments, no a priori sample size calculation was performed, and all experiments were performed in a non-blinded manner.

### 4.5. MTT Assay

Cellular viability was assessed through an MTT test [56] on OECs grown into 96-multi-well plates. In detail, 3-[4,5-dimethylthiazol-2-yl)22,5-diphenyl]tetrazolium bromide (MTT; Sigma, Kawasaki, Japan) was added to each multi-well at a final concentration of 1.0 mg/mL and incubated for 2 h in a CO_2_ incubator. Subsequently, isopropanol/SDS, an MTT solvent, was added, and cells were agitated on an orbital shaker for 15 min. The optical density was evaluated at λ = 570 nm with a microplate spectrophotometer reader (Cary 50 MPR microplate reader—Varian© 237, Palo Alto, CA, USA). Four replicates were carried out for each experimental condition, and results were expressed as the percentage MTT reduction in the control cells. 

### 4.6. TUNEL (Terminal Deoxynucleotidyl Transferase dUTP Nick End Labeling) Test

Dead cells were counted by staining with the Click-iT™ TUNEL Alexa Fluor™ 488 Imaging assay (Thermo Fisher Scientific©, Waltham, MA, USA), based on the incorporation of the modified nucleotide deoxyuridine triphosphate (dUTPs) at the 3′-OH ends of fragmented DNA by the enzyme terminal deoxynucleotidyl transferase (TdT). OECs were cultured on glass coverslips and exposed to LPS in the presence of EPA, DHA, EPA-EA, and DHA-EA for 24 h. Cells were fixed with 4% PFA, permeabilized with 0.25% Triton X-100, and processed following the manufacturing instructions. Coverslips were scanned with a Nikon Ti-Eclipse inverted microscope (Nikon©, Tokyo, Japan) equipped with a Plan Apochromat lambda 60×/1.4 oil immersion lens (Nikon©, Tokyo, Japan) and data acquired with NIS-Elements AR (Advanced Research) software (version 4.60) (Nikon©, Tokyo, Japan). TUNEL-positive cells were counted with the “CellCount” option in the NIS-Elements AR v4.60 software (Nikon, Tokyo, Japan), adjusting pixel threshold, size, and circularity, and reported as TUNEL+ nuclei/total cell number (DAPI+ nuclei) × 100 [59].

### 4.7. Mitochondrial Health Assay

Mitotoxicity and cytotoxicity in all OEC experimental conditions were detected by using the HCS Mitochondrial Health Kit (Thermo Fisher Scientific©, Waltham, MA, USA). Cells were plated into glass coverslips and exposed to LPS in the presence of EPA, DHA, EPA-EA, and DHA-EA for 24 h (three glass coverslips were carried out for each experimental condition). Briefly, following manufacture instructions, cells were incubated for 30 min with MitoHealth reagent, which accumulates in the mitochondria of living cells proportionally to the mitochondrial membrane potential, and with Image-iT DEAD™ Green™ stain, an impermeant dye to healthy cells that forms highly fluorescent dye-DNA complexes if plasma membrane integrity is compromised. Then, OECs were washed with PBS and fixed with 4% paraformaldehyde (PFA); the blue-fluorescent dye nuclear segmentation 4′,6-diamidino-2-phenylindole (DAPI) (Thermo Fisher Scientific©, Waltham, MA, USA) was used to visualize the nuclear compartment. Coverslips were visualized with a Nikon Ti-Eclipse inverted microscope through a Plan Apochromat Lambda 60×/1.4 oil immersion lens (Nikon©, Tokyo, Japan), and images were acquired with NIS-Elements AR v4.60 software (Nikon©, Tokyo, Japan). The fluorescence was quantified by analyzing the mean intensity of each channel from at least ten multiple regions of interest (ROI) normalized to the background by using the NIS-Elements AR (Advanced Research) software (version 4.60), as previously described by La Cognata et al. [59].

### 4.8. Immunocytochemistry

Immunocytochemical procedures to detect the expression of IL-10, an inflammation marker, vimentin, and GFAP, both cytoskeleton proteins, were applied to the cells incubated for 24 h in all the different experimental conditions. Cells were fixed by 4% PFA in 0.1 M phosphate-buffered saline (PBS) for 30 min. After washing in PBS, the cell membranes were permeabilized with 5% normal goat serum (NGS) in PBS containing 0.1% Triton X-100 (PBS-Triton) at room temperature for 15 min and then incubated overnight at 4 °C with the following primary antibodies: polyclonal antibody anti-IL10 (1:400, AbCAM, Waltham, MA, USA), mouse monoclonal antibody against vimentin (1:50, DAKO M0725, Santa Clara, CA, USA), and polyclonal antibody against GFAP (1:1000, DAKO, Z0334, Santa Clara, CA, USA). Secondary antibodies Cy3 anti-rabbit and Cy3 anti-mouse (1:500, Jackson Immunoresearch, Cambridge, UK) were used to visualize IL-10 and vimentin antibodies, respectively. Fluorescein Isothiocyanate (FITC)-conjugated goat anti-rabbit IgG antibody (diluted 1:200; Jackson Immunological Research Laboratories Inc., West Grove, PA, USA) was used to visualize GFAP antibody. OECs were incubated with the secondary antibodies for 1 h at room temperature and in dark conditions. The immunostained coverslips were analyzed with a fluorescence microscope (Carl Zeiss©, Jena, Germany), and images were captured with the Axiovision imaging system (Zen3.6-Blue edition). In all cell cultures in which primary antibodies were omitted, no specific staining was observed.

### 4.9. Immunostaining Quantification

The FIJI-ImageJ (1.52v software) measure tool (NIH, Bethesda, MD, USA) was used to carry out the immunostaining quantification. From each group, at least three samples were investigated at each time point. From each sample, three digital photomicrographs were randomly selected, and from each one, up to seven immunofluorescent cells were analyzed. Values were derived from the average grayscale intensity. The integrated density, the cell area, and the mean fluorescence of the selected cells were evaluated. At least three replicate measurements were performed for each capture region. The same procedure was applied to three different background areas around the selected cell. Then, the Corrected Total Cell Fluorescence (CTCF) was calculated using the following equation: CTCF = integrated density (cell area × background mean fluorescence).

### 4.10. Statistical Analysis

All experiments were repeated, at least in triplicate. Statistical analysis was performed using GraphPad Prism 9.0 (GraphPad Software, La Jolla, CA, USA). For immunofluorescence experiments, CTCF values are reported as mean ± SD. For TUNEL and MitoHealth/Dead green staining, values are reported as mean ± SEM. Differences between samples were assessed using a One-way analysis of variance (One-way ANOVA) followed by a post hoc Dunnett’s test. Significance was defined as follows: ** *p* < 0.01; *** *p*< 0.001; **** *p* < 0.0001 vs. CTR; # *p*< 0.05; ## *p*< 0.01; ### *p*< 0.001; #### *p*< 0.0001 vs. LPS.

## 5. Conclusions

In this study, a comparative analysis of the anti-inflammatory effects of EPA, DHA, and the corresponding *N*-ethanolamides in two different concentrations on typical olfactory glial cells, OECs, exposed to LPS-induced inflammation was reported. 

Our results show that all the tested compounds are neither cytotoxic nor mitotoxic at the tested concentrations. Indeed, the amide derivatives stimulate mitochondrial activity with an improvement in their metabolism. They also exert protective effects on OECs exposed to LPS by slowing down the cell death progression, reducing the expression of the IL-10 cytokine, and avoiding LPS-induced astrogliosis, as deduced by the decrease in the expression of cytoskeleton proteins such as GFAP and vimentin. All these data would contribute to attributing significant anti-inflammatory and neuroprotective activity to all the tested compounds, even if a more pronounced suppression of the inflammatory event was found for DHA-EA at both concentrations compared to EPA-EA in OECs exposed to LPS. 

In conclusion, our results, with the limitations associated with in vitro rather than in vivo experiments, highlight that EPA and DHA, both as free acids and *N*-ethanolamide derivatives, show high therapeutic potential in slowing the onset of the neurodegenerative diseases, so opening new prospects for therapeutic strategies. As a perspective in our future investigation, the protective role of the compounds here investigated against LPS-induced neuroinflammation would deserve a more in-depth study on olfactory glia cells in an in vivo model. 

## Data Availability

The data presented in this study are available in the article.
